# Evaluation of a technology-enhanced integrated care model for frail older persons: protocol of the SPEC study, a stepped-wedge cluster randomized trial in nursing homes

**DOI:** 10.1186/s12877-017-0459-7

**Published:** 2017-04-18

**Authors:** Hongsoo Kim, Yeon-Hwan Park, Young-il Jung, Hyoungshim Choi, Seyune Lee, Gi-Soo Kim, Dong-wook Yang, Myunghee Cho Paik, Tae-Jin Lee

**Affiliations:** 1 Department of Public Health Science at Graduate School of Public Health, Institute of Aging, Institute of Health and Environment, Seoul National University, 1 Gwanak-ro, Gwanak-gu, Seoul South Korea; 2 College of Nursing, the Research Institute of Nursing Science, Seoul National University, Daehakro 103, Jongno-Gu, Seoul South Korea; 3 Institute of Health and Environment, Seoul National University, 1 Gwanak-ro, Gwanak-gu, Seoul South Korea; 4 Youngsan University, College of Nursing, Yangsan Campus, 288 Junam-ro, 50510 Yangsan, Gyeongnam South Korea; 5 Department of Public Health Science at Graduate School of Public Health, Seoul National University, 1 Gwanak-ro, Gwanak-gu, Seoul South Korea; 6 College of Natural Sciences, Department of Statistics, Seoul National University, 1 Gwanak-ro, Gwanak-gu, Seoul South Korea; 7 Department of Public Health Science at Graduate School of Public Health, Institute of Health and Environment, Seoul National University, 1 Gwanak-ro, Gwanak-gu, Seoul South Korea

**Keywords:** Chronic care model, Geriatric care model, Long-term care, Technology, Stepped-wedge trials, Quality of care, Quality of life, Economics, Elderly, Process evaluation

## Abstract

**Background:**

Limited evidence exists on the effectiveness of the chronic care model for people with multimorbidity. This study aims to evaluate the effectiveness of an information and communication technology- (ICT-)enhanced integrated care model, called Systems for Person-centered Elder Care (SPEC), for frail older adults at nursing homes.

**Methods/Design:**

SPEC is a prospective stepped-wedge cluster randomized trial conducted at 10 nursing homes in South Korea. Residents aged 65 or older meeting the inclusion/exclusion criteria in all the homes are eligible to participate. The multifaceted SPEC intervention, a geriatric care model guided by the chronic care model, consists of five components: comprehensive geriatric assessment for need/risk profiling, individual need-based care planning, interdisciplinary case conferences, person-centered care coordination, and a cloud-based information and communications technology (ICT) tool supporting the intervention process. The primary outcome is quality of care for older residents using a composite measure of quality indicators from the interRAI LTCF assessment system. Outcome assessors and data analysts will be blinded to group assignment. Secondary outcomes include quality of life, healthcare utilization, and cost. Process evaluation will be also conducted.

**Discussion:**

This study is expected to provide important new evidence on the effectiveness, cost-effectiveness, and implementation process of an ICT-supported chronic care model for older persons with multiple chronic illnesses. The SPEC intervention is also unique as the first registered trial implementing an integrated care model using technology to promote person-centered care for frail older nursing home residents in South Korea, where formal LTC was recently introduced.

**Trial registration:**

10.1186/ISRCTN11972147

## Background

Reforming healthcare delivery to meet the complex care needs of an aging population is a top policy agenda in most developed countries [1, 2]. The healthcare cost of caring for older adults is high and consistently increasing, yet the quality of care for this population is often suboptimal, which can contribute to low health outcomes and well-being [1, 3]. Provision of person-centered coordinated care for older people with chronic conditions is known to be the key to care delivery reform, yet implementing such an innovative chronic care model (CCM) is challenging [1, 4, 5].

Some studies have reported positive effects of CCMs on quality of care, health outcomes, and/or satisfaction, although others have not [5–7]. Several gaps in implementing such models exist. First, many CCMs target a single chronic disease [5, 6], yet the majority of older adults have multiple chronic conditions [1, 3]. It is important to provide preventive care through CCMs in community primary care settings [7], yet CCMs are still relevant and needed even more for frail older people at long-term care settings, as they are in more complicated situations and often have more service use. In contrast to the CCM approach, several geriatric care models at nursing homes have also been proposed, but they have often targeted only certain disease groups (e.g., dementia), conditions (e.g., delirium, pressure ulcers), outcomes (e.g., adverse drug events, hospitalization), or care workers (e.g., team building, communication) [8]. Such approaches may be effective in yielding the intended positive changes in the specific problem-areas identified, but in practice, a model with a more whole-person approach at the system level is needed. Only one published study was found that evaluated such an intervention, by Boorsma et al. [9]: a multidisciplinary integrated care model using comprehensive geriatric assessment (CGA) improved quality of care in Dutch residential care facilities. The study was claimed to be CCM-inspired, but the linkage was not clear.

Further evidence is necessary on a CCM for older adults with complex chronic conditions; this study aims to address this gap. We developed an integrated care model guided by a CCM for older nursing home residents, named Systems for Person-centered Elder Care (SPEC). Unlike the Boorsma study [9], we combined the elements of a CCM with evidence-based practice in improving care delivery to create SPEC, an integrated platform tailored to long-term care settings in South Korea. In addition, SPEC is a technology-enhanced intervention using a prototype information and communication technology (ICT) system specially promoting an individualized care planning and monitoring process. Another unique aspect of this study is the proposed CCM model will be tested at nursing homes in South Korea, an Asian country where caring for older people is traditionally the responsibility of family members, but a formal LTC system was recently introduced [10], unlike countries in North America and Europe.

### Objectives

The aim of the study is to evaluate the effectiveness of a technology-enhanced, multidisciplinary, integrated care management model named Systems for Person-centered Elder Care (SPEC). The primary research question is whether the SPEC model (intervention program), a theory-driven, technology-enhanced, integrated care model, is effective for improving quality of care, compared to care reflecting the current practice pattern in nursing homes in South Korea. We hypothesize a person-centered, integrated, multidisciplinary care model will improve the quality of nursing care, which will in turn promote the quality of life of older residents. To evaluate the proposed model, we will conduct a clustered randomized control trial (RCT) for older adults residing in nursing homes, and the model will be implemented at the nursing home (cluster) level. We will also conduct economic and process evaluations.

## Methods/Design

### Study design

**Fig. 1:**
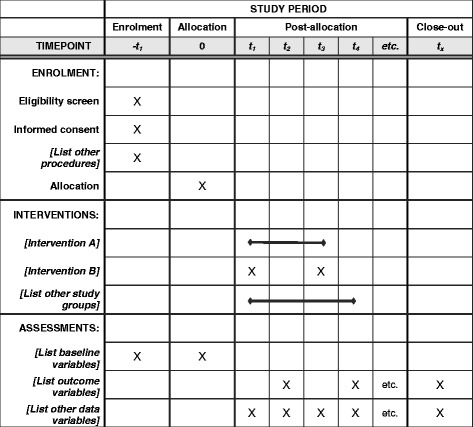
The schedule of enrollment, interventions, and assessments of the SPEC intervention. SPEC: Systems for Person-centered Elder Care Elder Care

Systems of Person-centered Elder Care (SPEC) is a multicenter, prospective, unidirectional cross-over cluster randomized control trial; older adults residing in ten participating nursing homes (the clusters) receive the intervention after a control period (Fig. 1). By adopting an incomplete stepped-wedge design [11], the SPEC intervention is sequentially rolled out to the nursing homes over five different time periods. After completion of enrollment of the ten nursing homes, they are randomly assigned into one of the five staring points, and the intervention is sequentially rolled out every three months to two nursing homes in pre-assigned random order. Older adults in each participating nursing home receive the intervention for approximately six months following a three-month control period during which the usual care (the comparator) continues to be provided. A total of four measurements will be conducted in each home, including a baseline measurement. The overall study period will be approximately 21 months. The trial is prospectively registered at ISRCTN. This protocol is reported following the SPIRIT guidelines.

### Study setting and participants

The SPEC study is being conducted at 10 nursing homes that have agreed to participate, located in the major provinces in South Korea. The participating nursing homes have been designated as long-term care institutions for beneficiaries of the public long-term care insurance (LTCI) for the elderly, a mandatory social insurance operated by the National Health Insurance Service in Korea [12]. In order to deliver services to LTCI beneficiaries, the nursing homes must meet certain regulations for staffing, physical facilities, and operations, required by law for the LTCI [12]. Nursing homes that have participated in any other intervention studies will be excluded.

Below are the inclusion and exclusion criteria of residents and staff participating in the study, and they are broadly defined. All adults aged 65 or older who reside at the participating nursing homes during the study period and who agree to participate are eligible to participate except those meeting following conditions: older adults staying at a participating nursing home for less than 7 days, older adults who are terminally ill or comatose, and older adults who are incapable of participating. As for staff, the inclusion criteria for study participation are as follows: working at a study nursing home during the study period, being involved in the care of the participating older residents, and agreeing to participate. Study participation is voluntary, and participants can withdraw from the study anytime they want.

### Intervention: The SPEC Model

#### Theoretical Rationale

This study is a stepped-wedge cluster randomized trial to evaluate the SPEC model, a technology-enhanced, integrated care model for frail seniors in long-term care facilities in South Korea. As a person-centered, multidisciplinary, integrated care-management model enhanced by technology, the SPEC model targets older residents at risk for common geriatric conditions and problems. The SPEC model is guided by the chronic care model (CCM) [13] and the CCM-inspired multidisciplinary integrated care (MIC) model for residential care homes [14]. The key tenet of the SPEC model is that the quality of care for frail older adults can be improved by implementing an innovative, person-centered system for delivering care rather than focusing on treatments of individual diseases and/or particular health conditions respectively.

Wagner et al. [13, 15] theorized how to improve chronic care and identified the following elements of the CCM: organizational support for and commitment to chronic care, decision support, delivery system redesign, self-management support, community resources. Guided by Wagner’s CCM, we constructed five key components of the SPEC model (intervention program) as follows: comprehensive geriatric assessment (CGA) for need/risk profiling, individualized need-based care planning (CP), interdisciplinary case conferences (ICCs), care coordination (CC), and ICT tools (ICT). The SPEC model was further developed by our research team, which has expertise in geriatric care models and the long-term care system in South Korea; the model was refined based on a literature review of existing studies, consultations with academic and practice experts, feedback from field staff, and also a pre-test of components of the SPEC intervention. Details of the SPEC intervention are summarized in Table 1.

**Table 1:** Descriptions of the SPEC intervention: Components, theoretical rationale, and implementation information

Component	Theoretical Rationale [Elements Comparable to Wagner’s CCM]	Outcomes	Providers/Participants	Place/Time	Dose
1. Comprehensive Geriatric Assessment (CGA)	[Decision support] CGA and CGA-based need/risk profiling of triggered common geriatric problems using a decision-support tool can promote a whole-person approach.	- CGA-based risk profile including key functional scales	- By the RN-SW pair at each participating nursing home	- At a nursing home - Right after the assessment by external assessor at T1 is completed	- At least one time for each of the participating residents - Anytime needed (e.g., condition change of residents)
2. Individualized Need-Based Care Planning (CP)	[Delivery-system design/self-management support] Individualized need-based CP using standardized care protocols and checklists by onsite SPEC coordinator-led interdisciplinary care teams, along with input from the resident/family regarding preferences and choices. These approaches can promote shared goal-setting and resident/family’s engagement in care delivery.	- Individualized, written care plans with goals, timeline, and to-do list in checklist form for each member of the care team - Resident/family input	- By the care team led by the RN-SW pair at each home	- At a nursing home - Right after the CGA is done	- At least one time for each of the participating residents - Anytime plan change is needed
3. Interdisciplinary Case Conferences (ICCs)	[Delivery system design/self-management support] Formal face-to-face interdisciplinary team meetings provide an opportunity for the care team to better understand complex case needs and develop a well-coordinated, targeted care plan; an optional intervention component due to limited financial and human resources.	- Same as above - Possibly a more coordinated action plan	- By the care team led by the RN-SW pair at each home and facilitated by the SPEC consultant	- At a nursing home - Once a month on average and when a relevant case is found	- At least once a month
4. Care Coordination (CC)	[Community resources] Coordination of care using tailored reports based on CGA/CP between care staff (administrators and direct care members), families, and contracted physicians/medical institutions can facilitate communication and promote quality of care.	- Administrative decision-making, order change, and/or provision of information to residents and family, if needed - Better collaboration with community resources and strengthening community linkages (e.g., contracted doctor, clinic, etc.)	- The RN-SW pair facilitated by the SPEC consultant	- When CGA and CP are done - Anytime needed	- At least once a month when CGA and CP are done - Anytime needed
5. ICT tool: the SPEC information system	[Clinical information systems] A cloud-based online ICT system can promote communication between care team members and also between care teams and the research team. The system makes it easy to store and track resident data and generates various tailored reports. It also provides resources for care providers/managers. KakaoTalk, a free instant message and phone call service in South Korea, is also actively used for communication throughout the program implementation and evaluation.	- Improved quality of CGA and CP using information system - Improved quality of documentation and monitoring of CGA, CP, and MCC	- The onsite SPEC coordinators facilitated by the SPEC consultant	- A server managed by a server manager is located at the SPEC research center - A help desk service is also provided - Users at each home can access the system anytime - E-accessed via desktop, tablet, and/or cell-phone	- Anytime needed

#### Components of SPEC

1. *Comprehensive geriatric assessment* (*CGA*) *for need/risk profiling:* Unlike traditional studies applying CCMs that target a single chronic disease, SPEC targets older people with complex conditions, for which CGA is essential for need/risk profiling [9, 14]. Through CGA, care teams are able to identify the multidimensional, and sometimes interconnected, needs of residents, which can promote a whole-person approach. We adopted interRAI LTCF [16], a widely used CGA tool in which evidence-based need/risk profiling algorithms are embedded; thus, by completing the assessment, the assessors in the care team can obtain a list of key functional scale results and a list of triggered need/risks tailored for each resident. These results, taken together, work as a decision-support tool for nursing home staff to profile needs/risks of their residents.
2. *Individualized need-based care planning* (*CP*)*:* Care planning is known as the foundation on which individualized and coordinated care can be organized and delivered, which can have positive impacts on quality of care [16–18]. Based on information from CGA reports, the interdisciplinary care team in each nursing home, under the leadership of a SPEC coordinator team consisting of a nurse and a social worker, develops a care plan for each resident with input from the resident/family regarding their preferences and choices in order to promote their engagement in the care-planning process. To support CP, the SPEC program provides the interRAI LTCF’s clinical assessment protocols (CAPs) book [19] and also a set of checklist forms with possible action points for the triggered risks (a problem list). The action points in the checklists are activities for assessment, management, evaluation, and/or coordination to decrease the identified risks and/or promote the strength of older adults. The checklists are based on the interRAI CAPs; but the SPEC research team, through literature review and consultations from academic and clinical experts, has localized them to meet the needs of Korean nursing homes. The checklists are uploaded on the SPEC system, a prototype, cloud-based ICT tool that will be explained later; each care team chooses relevant action items from the template-type checklists using their clinical judgement and considerations of unique resident and facility needs. Care teams can also add new items that are not in the template checklists. To promote person-centered care, once a draft care plan is developed, it is reviewed and discussed with residents and/or family members, updated, and confirmed, reflecting residents’ needs and preferences; this practice has rarely existed in nursing homes in Korea, although it may be common or accepted as a standard in Western countries.
3. *Interdisciplinary case conferences* (*ICCs*)*:* Case conference is a goal-oriented, systematic approach, characterized by exchanging ideas and opinions among team members on certain care problems and developing solutions for the problems, on which the team agrees and acts collaboratively [20, 21]. In the SPEC model, the care team can have optional interdisciplinary case conference meetings for the cases of older people who are newly admitted, at high risk, and/or have complex care needs [20, 21]. In-depth discussions between care team members are necessary for delivering care to complex cases in effective and coordinated ways. ICCs are not a new concept, but almost all the nursing homes participating in our study admitted that either they did not do ICCs at all due to limited resources, or they did ICCs, but they were somewhat ineffective and superficial. In the SPEC model, we support the care team in doing an informed ICC by providing information in each resident’s profile from the CGA and the tailored CP. Guidelines for the ICC were developed based on a review of literature, existing guidebooks for case conference models, and multidisciplinary expert feedback as well as the research team’s own expertise.
4. *Care coordination* (*CC*)*:* Care coordination is a well known critical factor for delivering quality care for people with complex chronic needs [15, 16, 18]. The SPEC program focuses on improving communication and engagement between the home care team and contracted physicians and family of older residents in the community using evidence-based reports, reflecting the context of long-term care delivery in South Korea. In order to promote communication among stakeholders, the SPEC model provides tailored reports to three targeted stakeholders: nursing home administrators, contracted physicians, and family members. The reports are based on CGA and CP results. The care team uses the report to facilitate communication and coordination with those stakeholders. Nursing home administrators receive an institutional-level summary report on the resident’s profile and care needs, and the report also includes benchmark statistics. We also provide to the contracted physicians a dashboard-type report giving a summary of prevalent health and functional issues and a list of high-risk groups for major CAPs. Lastly, the report for family members informs them in a relatively simple, summarized way of the current heath and functional status of the resident, care goals, and key activities.
5. *Information and communications technology* (*ICT*) *tool:* The last component of the SPEC intervention is an ICT tool. Korea is one of the most wired countries in the world [22]. The computerized SPEC assessment and management system (SPEC system), as a prototype, cloud-based ICT tool, was developed to take advantage of this. The SPEC system supports and tracks the entire needs-assessment and care-management process: CGA, care planning, report generation, and look-up of resource materials. The SPEC data center authorizes users to access the SPEC server using a desktop computer, tablet PC, and/or cellphone with a proper password, and the user can record their assessment data; all research data are stored in the server in the SPEC data center rather than the personal computer of users. We also actively use KakaoTalk, a free, mutifaceted mobile messaging application for sharing texts, images, videos, etc., through which SPEC consultants and data center people provide care teams in each facility with frequent and immediate off-site support. The research team also uses the free message service to check the status of the intervention and data collections and to deliver information (e.g., reminders for scheduled education sessions, etc.). Further details of the SPEC intervention are summarized in Table 1.

### Procedure

The intervention procedures are as follows: the SPEC model uses a SPEC consultant who understands the philosophy/vision of the model and is also well versed in specific aspects of the model. As a circulating consultant, the SPEC consultant trains and empowers the staff and facilitates the whole implementation of the SPEC model in the participating nursing homes. In each home, an onsite SPEC coordinator team consisting of a nurse-social worker pair trained by the SPEC consultant conducts the internal implementation process of the model. The coordinator team conducts CGAs and develops individualized care plans based on a set of protocols in collaboration with interdisciplinary team members within and outside of the nursing home. The interdisciplinary team-developed care plans are reviewed, modified/negotiated, and confirmed by older residents and their family. CGAs and CP are redone whenever needed (e.g., condition changes, upon request by residents, etc.).

In addition, for complex, high-risk cases or newly admitted residents, interdisciplinary care conferences (ICCs) are held for more in-depth team discussion on care planning and coordinated delivery. In the ICC, CGA reports and preliminary care plans for the case are discussed and refined. During and after the CP and/or ICCs, the onsite SPEC coordinator team coordinates health care for residents with contracted physicians and also other community health and social care organizations, if needed. They also use family reports created based on CGAs and the CP/ICCs for communication with and support from family members. Ongoing care coordination and care management is supported by a prototype, cloud-based ICT tool; all data and/or reports from the CGAs, CP, and ICCs for each resident are recorded, updated, stored, and shared. Printable, downloadable online worksheets to track progress and completion of the planned care activities outlined in the care plans are provided. The onsite coordinator team uses various reports that can be generated for staff, administrators, contracted community physicians, and/or residents/families. Resources for nursing home staff include evidence-based best-practice guidelines, resident education materials, video links, etc. Lastly, all relevant participants in the SPEC intervention have mobile phones, so they use KakaoTalk, a free mobile messenger app that allows no-charge phone calls, texting, and video chats. KakaoTalk is constantly used between nursing home staff members, and also between nursing home staff and SPEC teams for the purposes of coaching, troubleshooting the system’s operation, and sending reminders.

Various implementation strategies are used, tailored to the needs and context of each home. Core strategies include information sessions for top-level nursing home administrators, a kick-off meeting for interventions, education and training sessions for CGA and CP, motivational interviews with the onsite coordinator team by the SPEC consultant, and a steering group at each participating home. The extent of protocol adherence and intervention implementation is regularly monitored.

### Comparator

During the control period, older adults receive the usual care by staff at each participating nursing home (cluster), which is identical to the usual practice. While “the usual practice” may not be identical across homes, care staff generally do some resident assessments, though neither CGA with decision support nor CGA results- (evidence-)based ICCs tend to be conducted in a systematic way. CP is often neither individualized nor documented properly. The participation of older residents and/or their family members in the care planning process is very limited. The execution of care plans is left to the discretion of care staff of each home, following their existing practice patterns. Any other concomitant care or interventions that may affect trial outcomes are not permitted in the participating nursing homes during the current trial.

### Outcomes

Quality of care is the primary outcome, and several secondary outcomes including quality of life will be measured. We have also included process measures and staff-/organization-related measures as complementary. All outcomes and their measurement plans are summarized in Table 2, except the process evaluation measures, which are in Table 3.

**Table 2:** Overview of the outcome variables, measures, and observation points

Variable	Data Source/Instrument	Measurement Points	Unit of Analysis
Primary Outcomes			
Quality of Care	interRAI LTCF quality measures (composite score)	T0 (baseline), T1, T2, T3	Resident
Secondary Outcomes: Patient-Related			
Quality of Care	interRAI LTCF quality measures (individual score)	T0 (baseline), T1, T2, T3	Resident
Care Needs	Number of triggered clinical action points of interRAI LTCF	T0 (baseline), T1, T2, T3	Resident
Functional Health	Bathel index; Mini-Mental Status Examination; Patient Health Questionnaire	T0 (baseline), T1, T2, T3	Resident
Quality of Life	EuroQol(EQ)-5; interRAI HRQoL; interRAI self-reported QoL (SQoL)	T0 (baseline), T1, T2, T3; T1 & T3 (SQoL)	Resident
Patient Satisfaction	Client Satisfaction Questionnaire	T1 & T3	Resident
Health Care Utilization	Hospital admissions; emergency department visits	Every three months	Resident
Costs	Questionnaire on the cost; direct and indirect costs	Every three months	Resident
Other Outcomes: Care Staff/Organization-Related			
Empowerment	Psychological Empowerment Instrument	T1 & T3	Nursing Home
Communication Satisfaction	Communication Satisfaction Questionnaire	T1 & T3	Nursing Home
Organizational Commitment	Organizational Commitment Questionnaire	T1 & T3	Nursing Home
Job Satisfaction	Job Satisfaction Scale	T1 & T3	Nursing Home
Technology/Innovation Acceptance	Modified Technology Acceptance Model Questionnaire	T1 + 1 month & T3	Nursing Home

**Table 3:** Process evaluation on intervention and implementation strategies

Topic	Data Source/Analysis	Measurement Points	Unit of Analysis
Recruitment of cluster	[Qualitative analysis] - Measurement from documentation of recruitment & allocation process by research team	During recruitment of clusters	Nursing home
Delivery to clusters	[Quantitative analysis] - Measurement of intervention fidelity across its components: in-service training; care planning; case conferences with assistant & case conferences without support; coordination of care; supporting & tracking through ICT	During and after each component	Nursing home & Resident
	- Measurement of receipt in target population from the cloud-based SPEC computerized system	T2, T3	Resident
	[Qualitative analysis] - Measurement from interviews with onsite SPEC coordinators in charge of case management about what intervention is delivered and why	After T3	Nursing home
Response of clusters	[Quantitative analysis] - Measurement of standardized questionnaire items sent to participants along with other follow-up measurements	After T3	Nursing home
	[Qualitative analysis] - Measurement from documents and interview data about target population’s experience of and response to the intervention.	During and after each component	Nursing home
Recruitment and reach-in of individuals	[Quantitative analysis] - Measurement from documents and participant list	T0	
	- Quantitative comparison of those receiving vs. not receiving the intervention	T0, T1, T2, T3	Nursing home & Resident
	[Qualitative analysis] - Measurement from interviews with those who recruited the participants	During and after each component	Nursing home & Resident
Response of individuals	[Quantitative analysis] - Measurement from standardized questionnaire of individuals’ perceptions of the intervention and uptake of trial components. (TAM, questionnaire for process evaluation)	T1 + 1 month; after T3	Nursing home
	[Qualitative analysis] - Measurement from observational and interview data about target population’s experience of and response to the intervention (documents, web data, telephone calls, audiotape of case conferences, etc.)	During and after each component	Nursing home
Context	[Quantitative analysis] - Measurement from project manager’s documents and from standardized questionnaire to assess organizational and structural characteristics of nursing home	Before T1; after T3	Nursing home
	[Qualitative analysis] - Measurement from policy documents or interviews (semi-structured interviews to assess “care as usual” at baseline and at end of study)	Before T1; during and after each component; after T3	Nursing home
Implementation strategies	[Quantitative analysis] - Measurement of quantitative data from web and documents	During and after each component	Nursing home
	[Qualitative analysis] - Measurement from project manager’s documents and interview data about target population’s experience of and response to the facilitation strategies	During and after each component	Nursing home

#### Primary outcome measure

The primary outcome measure of the study is *quality of care* (*QoC*)*,* measured by a composite score of quality indicators (QIs) in the interRAI LTCF, which has good psychometric properties [23], similar to the primary outcome measures of the MIC intervention in resident care facilities in the Netherlands [14] and the European Services and Health for Elderly in Long-TERm Care (SHELTER) study [24]. The outcomes will be measured four times: at T0 (baseline), T1 (3 months; the end of observation period), T2 (6 months; 3 months after the beginning of the intervention), and T3 (9 months; the end of the intervention, 6 months after its start). Resident outcome data will basically be collected every three months by external nurse assessors trained by the SPEC research team.

#### Secondary outcome measures

*QoC*, measured by individual interRAI QIs, is a secondary outcome measure to observe the effects of the SPEC intervention on quality in various health and function domains separately, assuming the impact of the program may not be the same across the domains.

*The extent of care needs* is measured with the number of total triggered CAPs in four categories: functional performance, cognition/mental health, social life, and clinical issues [19]. We will examine the change in the number before and after the program implementation. Some CAPs may have too low a prevalence in Korean nursing homes, which would threaten their validity, so we will remove them from the final set of CAPs for the analysis.

*Functional health* will be measured by three measures (the Modified Bathel index, Mini-Mental Status Examination, Patient Health Questionnaire). The Bathel index is used to measure performance of physical functions in activities of daily living. We will adopt the modified version by Shah et al. [25], which has demonstrated high inter-rater reliability (0.95) and test–retest reliability (0.89) as well as high correlations (0.74–0.8) with other measures of physical disability [26]. The Mini-Mental Status Examination (MMSE) [27] is used to measure cognitive impairment using a 30-point questionnaire. The Korean version of MMSE (K-MMSE) has good sensitivity (0.70-0.83) and convergent validity (0.78) [28]. The PHQ-9 is a depression module of the PHQ (Patient Health Questionnaire) developed by Spitzer et al. [29] and translated into Korean by Han et al. [30]. The Korean version of the PHQ-9 has good internal consistency (0.88) and convergent validity (0.74).

The *quality of life* (QoL) of older nursing home residents will be measured with the Korean version of the EQ-5D-5 L. The EQ-5D, developed by EuroQol Group, is a measure of health-related QoL that has five items, and each item measures different dimensions of health–-mobility, self-care, usual activities, pain/discomfort, and anxiety/depression [31]. The EQ-5D-5 L will be adopted because 5 L is known for having better sensitivity and a lower ceiling effect than other versions of EQ-5D in Korea [32]. General QoL of residents will be measured with the interRAI Self-Reported QoL (interRAI SQoL) [33].

*Patient satisfaction* will be measured with the K-CSQ (Korean Client Satisfaction Questionnaire) [34]. The original CSQ developed by Larsen et al. [35] has six different forms, and CSQ-8, consisting of 8 items, is most widely used in consumer or client satisfaction studies. The K-CSQ by Kim et al. [34] is a translated and modified version of CSQ-8 to measure the satisfaction of nursing home residents in Korea; good psychometric properties have been reported.

As for *health care utilization*, costs will be collected from a societal perspective and consist of medical costs and intervention costs. Medical costs will be calculated by multiplying the healthcare utilization and unit costs. Other healthcare utilization data includes the number of physician visits, length of stay, service use in the facilities, and time consumption. Nationally representative data—such as the National Health Insurance database [36] and Korea Health Panel Survey data [37]—will be used to calculate the unit costs. Intervention costs that occur while operating the SPEC program will also be calculated. Productivity costs are to be excluded, taking into account the old age and frail health status of the residents.

#### Other measures: Staff-level & organization-related measures

Other outcome measures include care staff-level and organization-related measures, for which participating nursing staff members will directly answer a set of survey questionnaires.

*Empowerment* of the nursing home staff members will be measured with the Psychological Empowerment Instrument (PEI), which was developed by Spreizer [38] and translated into Korean and modified by Ahn [39]. The PEI is composed of 4 domains (meaning, competence, self-determination, impact), and each domain has 3 items for a total of 12 items. The original instrument’s 7-point scale was modified into 5-point scale by Ahn [39], and its Cronbach’s α was 0.898.

*Communication satisfacti*on will be measured with the Communication Satisfaction Questionnaire (CSQ) developed by Downs & Hazen [40], translated into Korean and modified by Lee [41], and optimized to be used in health care settings by Park [42]. In Lee’s study [41], the original 40 items (8 domains, 5 items in each domain) of the instrument were modified into 24 items (3 items for each of the 8 domains).

*Organizational commitment* refers to employees’ identification with and attitudes toward the organization they are affiliated at, and also strength of involvement and relationship with the organization [43], which will be measured by the 15-item Organizational Commitment Questionnaire (OCQ) that was developed by Mowday et al. [43] This study has adopted the OCQ as translated and modified by Park [44] for use at long-term care settings in Korea.

*Job satisfaction* is measured with the 20 items on the self-rated Korean Minnesota Satisfaction Questionnaire (K-MSQ) [45] to measure organizational commitment, which was a modified version of Park’s [46] translation of the short version of the MSQ [47]. The K-MSQ [45] is a version of the MSQ applied to health care settings; Cronbach’s α for the instrument was 0.795 for the extrinsic factors, 0.862 for the intrinsic factors, and 0.908 for the general factors.

*Technology acceptance* will be measured with the TAM questionnaire, which was developed based on the Technology Acceptance Model [48]. The original TAM is a 10-item, self-rated questionnaire using a 7-point Likert scale, and it consists of 4 domains as follows: behavioral intention, perceived usefulness, perceived ease of use, and subjective norm. No existing TAM questionnaire in Korean is relevant for nursing home settings in Korea, so we translated and modified the TAM for this study and pre-tested the instrument, which resulted in good reliability.

#### Process measures

It is challenging to evaluate a complex intervention like the SPEC, as the intervention itself combines various components into an integrated program. Thus, each component is distinct and independent, but at the same time, somewhat interconnected. In addition, the intervention is not implemented in a vacuum, but situated in the unique context in which the intervention is delivered [49, 50]. Thus, process evaluation is invaluable for understanding the background, integrated process, and results, and also can give insights into applying the same intervention to a different setting in the future [49]. The process evaluation will use a mixed methods design, in which quantitative and qualitative data will be collected using standardized questionnaires, case studies, and focus group interviews guided by open-ended questions. The data will cover the following topics: the process of recruiting the nursing homes, delivery of the intended program (interventions), responses of participating homes to the program, recruitment of and outreach to actual resident participants in each home, response of individuals, context of program delivery, and implementation strategies (Table 3).

### Sample size & recruitment

Sample size was calculated using the formula for incomplete stepped-wedge CRT designs [11]. The primary outcome of the trial is quality of care (composite score of QIs of the interRAI LTCF), and the expected intervention effects (proportion 1: 0.115, proportion 2: 0.182) were determined based on a study by Boorsma et al. [9], a similar intervention study. The ICC was also set to be identical to Boorsma et al.’s [9], which was 0.01. Unlike Boorsma, however, we modified the correlation matrix V of Hemming et al.’s [11] formula in two ways. First, in order to take into account that the primary outcome will be measured repeatedly on the same residents, we added a resident-specific random effect in the calculation of the correlation matrix; we assumed the correlation coefficient was 0.25 based on the ratio between the ICC and correlation coefficient used in Mutinga et al. [51]. Second, we also accounted for the fact that some QIs included in the composite QI score for the primary outcome were measured as the difference between two successive time points for the same individual in the same cluster. The calculation was done using R 3.2.4., assuming a total of 10 clusters where 2 clusters are randomized at each step.

Based on the power calculation, the required minimum cluster size for detecting the expected intervention effect on the primary outcome with an 80% power under alpha = 0.05 is *n* = 45. (If the outcome is measured separately at each time point, *n* = 33.) We aimed to recruit nursing homes with at least 67 beds based on two assumptions: that about 80% of residents in a public LTCI-certified nursing home in Korea would meet our trial inclusion criteria and agree to participate, and also that about 15% of the recruited older residents would drop over the course of our trial. These assumptions were based on our earlier national nursing home survey study [52] and also publicly available data on the characteristics of LTC residents [53].

In order to promote recruitment, the participating nursing homes were recruited between March and April, 2015. In-person information sessions organized by the PI and key research team members were held multiple times at a convenient time and place for nursing home administrators and staff members who showed interest in study participation for quality improvement purposes. The SPEC program is an institution-level intervention for quality improvement purposes, so we offer it to all older residents in participating homes who meet our recruitment criteria; and if they agree to participate, informed consent is obtained after a detailed explanation of the study. We allow new enrollments when an older adult is newly admitted in a participating nursing home and (s)he meets our trial criteria. New enrollments mainly occur when existing participants drop due to discharge or are transferred to other institutions or communities, etc., due to the high occupancy rate of participating homes. The new enrollment is not only for recruitment purposes but also for a practical purpose: the participating homes would like to assess and provide care management for their newly admitted residents.

### Randomization and blinding assignment of interventions

Using computer-generated random numbers, an allocation sequence for the recruited nursing homes (clusters) has been generated that complies with the statistical power calculations and the requirements of participating nursing homes. In each block, two homes has been randomly assigned. The random-sequence document is available neither to the enrolled nursing homes nor the participating older adults. In order to conceal the sequence, each nursing home was simply informed just one month prior to each home starting to recruit residents and get consent forms. None of the participating homes know the allocation sequences of either itself or others. A data manager independently has allocated the sequence and passed the results to the SPEC consultant enrolling the participating nursing homes. Outcome assessors and data analysts will be blinded after assignment to interventions. Outcome assessors will not be aware of the on-going intervention study. They will be responsible for data collection and entry in the electronic form. Data analysts will get the data without any identification information on the participating institutions and individuals. Blinding is not possible for trial participants and care providers in this study because it uses a stepped-wedge design where all participants receive interventions sequentially. Older residents will be informed they will receive an intervention upon consent, while outcome assessors and data analysts will be blinded until the end of the study.

### Data collections, management, and monitoring

Data collection will be conducted as outlined in Tables 2 and 3. Primary and secondary outcome data are being collected by trained external assessors who are also nurses with clinical knowledge about and experience with caring for older adults using the cloud-based SPEC system. For the external assessors, a one-day intense education program and a follow-up are offered: the program begins with an overview of the study, measurement plan, and responsibilities of the assessors. Then the instructors, who have extensive knowledge of measures and field data collection experience, provide an item-by-item, standardized, manual-based review of the study instruments, followed by a Q&A and discussion session with example scenarios. The later part of the education program consists of how to use the web-based data collection and storage program along with mobile data collection tools such as an iPad and/or cell phone. Along with a lecture session, a demonstration and practice session is provided using educational materials including detailed instructions and exercises. In order to become familiar with the cloud-based SPEC system, trainees are asked to do data entry and storage for several mock-cases, entering them into the system independently within a few weeks after the training session. To increase the reliability of assessment, double-assessment on a few random cases is also done by a pair consisting of an experienced assessor and a new assessor. On-going support for data collection will be provided via KakaoTalk, phone call and face-to-face meeting throughout the entire data collection process; and a helpdesk for the technical issues related to the use of the SPEC system is also provided.

Data of staff-level outcomes before and after the program implementation will be collected from nursing-home care staff members who are involved the SPEC program. Using double-blind methods, the staff members cannot be identified by the research team, which is a way to prevent bias in responses. Technology/innovation acceptance of care staff members will be measured using a survey instrument about one month from the first use of the SPEC system and then re-evaluated at the end of the intervention to compare the difference in the staff’s perceptions of technology use. A process evaluation will be conducted before, in the middle, and after each SPEC component is implemented. Qualitative interviews with the onsite SPEC coordinators in each home along with quantitative data on intervention fidelity will be collected.

To promote participant retention and complete follow-up, the SPEC consultant checks with the onsite SPEC coordinators regularly on the status of the intervention implementation in each site. Also, they are asked to report any issues with the participation status of older residents (e.g., transfer to hospital, death, etc.). If any discontinuation of participation occurs, a short-form discharge/discontinuation report is filled out immediately by the onsite SPEC coordinators, the nurse-social worker pair. Using the reports, the reasons for and patterns of discontinuation are monitored, and strategies to prevent such events are discussed in the weekly research team meeting.

### Data analysis plan

#### Effect evaluation

Baseline differences between clusters during the first control period will be tested using chi-square tests and ANOVA. The primary analysis will be the evaluation of the interventional effect of the SPEC program in nursing homes. The intervention effect on the primary outcome and secondary outcomes described above will be estimated using a multilevel regression analysis to account for the clustered structure [54]. We will use a generalized linear mixed effects model [54], which allows inclusion of both fixed (intervention, time) and random factors. In particular, two random effects will be introduced, one at the cluster (nursing home) level and the other at the individual (resident) level. The test statistic to assess any significant benefit of the intervention will be calculated. We will use two-sided p-values with alpha = 0.05 for level of significance. Compliance issues will be handled as in Jo et al. [55].

A secondary analysis will be conducted to adjust for the potential confounding effects of pre-randomization variables. The same generalized linear mixed effects model will be used to model the primary and secondary outcomes, adjusted for socio-demographic variables including age, sex, physical status, and cognitive status as well as an intervention indicator and time. We will also investigate the effect of the duration of the intervention by adding an interaction term between time and intervention as a fixed effect. Furthermore, subgroup analysis will be used to explore the subgroup-specific effects of the intervention on outcomes. The interaction effects of the intervention with dependency in activities of daily living and cognitive impairment will also be explored. When a statistically significant difference is found, the study population will be split to examine the results for each subgroup separately. We will also conduct an analysis of the intervention effects on facility-level pre-post measurements using a t-test. In addition, we will develop models to evaluate the impact of the program on resident-level outcomes while adjusting for facility-level factors. A *p*-value of 0.05 or less will be considered to be significant.

We will first conduct an effect analysis based on the intention-to-treat (ITT) principle [56], in which all older residents in the SPEC program will be included. Imputation methods will be implemented to handle missing data to enable ITT analysis [57]. We will allow new individuals to participate in the program at every time period; sensitivity analyses to assess the effect of attrition and inclusion of residents will also be conducted. The data monitoring committee (DMC) is composed of the PI, data managers, and statisticians; the committee is responsible for the data quality across the whole data-collection, analysis, and reporting process. The DMC is independent of the research-funding agency and has no competing interests.

#### Economic evaluation

A cost-effectiveness analysis will be conducted. The primary (quality of care) and secondary (e.g., Quality-Adjusted Life Years, QALYs) outcome measures will be used as indicators of effectiveness [58]. The QALYs will be calculated using health utility scores estimated by the Korean EQ-5D-5 L tariff [32]. In the analysis, incremental cost-effectiveness ratio (ICER)—defined as the difference in costs between the SPEC and usual care divided by the difference in the effect of the two types of care—will be assessed [59]. If the SPEC program is more costly and also more effective, we will calculate the incremental costs and effects. If the SPEC program is as effective as usual care, we will do the economic evaluation based on incremental costs only [60].

#### Process analysis

The goal of process evaluation of this study is to understand the context, integrated process, and results of the intended intervention program, and also can give insights into applying the same intervention to a different setting in the future [49]. Guided by the framework for designing process evaluation for cluster-randomized trials of a complex intervention [61], we aim to explain the potential differences between the expected and real outcomes, and provide insights on the facilitating and inhibiting contextual factors in program implementation as well as the intervention process, recruitment strategies, etc. [62]. Quantitative data will be analyzed using descriptive statistics, and qualitative data will be analyzed using content analysis, document analysis, etc.

### Ethics and dissemination

This study was approved by the institutional review board (IRB) of human subjects where the first author is affiliated (SNU IRB 1410/002-018). No critical protocol modifications have been made; and if they are, such changes will be communicated to all relevant parties, including the IRB, trial participants, the trial registry, journals, etc. In order to make sure the participation of older residents is voluntary, following the recommendation of the IRB, an adult attendant with no conflict of interest attends the consent-attainment process and co-signs the consent form to indicate the consent has been properly acquired. A password-protected electronic file including personal information, separate from the trial dataset, is saved in a password-protected computer in a locked room. None of the investigators or participating sites have financial or competing interests. Only the PI and those with the permission of the PI will have access to the final trial dataset. We plan to present the trial results in academic conferences, publish articles in peer-reviewed journals, and report to the trial registry when the analyses finish, regardless of the magnitude or direction of the effect. Authorship eligibility will follow the ICMJE guidelines, and we do not plan to hire professional writers.

### Trial status

The study has been rolled out to all ten nursing homes, and data collection is ongoing at the submission of this manuscript. Data analysis has not yet started.

## Discussion

As far as we know, this is the first registered trial to evaluate a CCM-based, ICT-enhanced geriatric care model for older people with multi-comorbidities in South Korea. We have developed and are evaluating the multifaceted SPEC intervention, implementing a multidisciplinary care management program. The program promotes person-centered care for older people through individualized CP based on CGA, and incorporates the preferences and choices of older residents and their families. We expect the SPEC model will support the nursing home staff to organize and deliver care in a more resident-centered way, which would primarily improve quality of care, which in turn would contribute to the health and well-being (quality of life) of older residents. The intervention will be tailored to each participating nursing home’s existing administrative routine, level of advancement in care planning, and management system, as well as to the needs and preferences of the identified older adults and/or their family members.

As for study design, this SPEC study adopted a stepped-wedge cluster randomized trial (SWCRT) design [11, 63, 64], which is appropriate for this study in several ways. First, the SWCRT design provides an opportunity for all participating nursing homes to get the intervention at different time-points during the intervention period. This was a critical point in the recruitment of the participating homes, which are resource-limited and busy in their day-to-day practice; they all wanted to get the intervention as a condition for participation. Second, the design is suitable for the research team, which, with its limited budget and manpower, would not be able to roll out the intervention to all ten nursing homes at the same time while ensuring the quality of implementation. Third, the study design allows an estimation of the intervention effects using both a between- and within-cluster comparison.

There are several anticipated challenges. The constraints in financial and human resources may decrease the fidelity of participating nursing homes to the implementation of the SPEC program. Along with these resource limitations, preparations for an anticipated mandatory quality inspection by the government [12], the top priority of nursing home administrators, also could weaken nursing homes’ commitment to the SPEC program. Other potential challenges include difficulty in engaging nursing home staff to learn and practice new knowledge and skills for conducting CGAs and CP along with using the new ICT system, as many nursing homes still rely on paper-based charting systems. Getting cooperation from frail older residents for data collection is another anticipated challenge. Along with several strategies described above to improve study fidelity, the study team will provide tailored guidance, support, and encouragement for each participating home, as the barriers and facilitators each home faces may differ. One possible strategy for retention of older residents is to decrease the burden of being interviewed for data collection by adjusting the data collection schedule and time. Similarly, the research team will adjust the time and place for education and evaluation at each home as much as possible within the given data collection schedules.

## Abbreviations

CC: Care coordination
CCM: Chronic care model
CGA: Comprehensive geriatric assessment
CP: Individualized need-based care planning
DMC: Data monitoring committee
ICC: Interdisciplinary case conference
ICT: Information and communication technology
LTC: Long-term care
LTCF: InterRAI Long-term care facilities
LTCI: Long-term care insurance
QALYs: Quality-adjusted life years
QI: Quality indicator
SPEC: Systems for person-centered elder care
SWCRT: Stepped-wedge cluster randomized trial
