# Erratum to: Evaluation of a technology-enhanced integrated care model for frail older persons: protocol of the SPEC study, a stepped-wedge cluster randomized trial in nursing homes

**DOI:** 10.1186/s12877-017-0495-3

**Published:** 2017-05-15

**Authors:** Hongsoo Kim, Yeon-Hwan Park, Young-il Jung, Hyoungshim Choi, Seyune Lee, Gi-Soo Kim, Dong-wook Yang, Myunghee Cho Paik, Tae-Jin Lee

**Affiliations:** 1 Department of Public Health Science at Graduate School of Public Health, Institute of Aging, Institute of Health and Environment, Seoul National University, 1 Gwanak-ro, Gwanak-gu, Seoul, South Korea; 2 College of Nursing, the Research Institute of Nursing Science, Seoul National University, Daehakro 103, Jongno-Gu, Seoul, South Korea; 3 Institute of Health and Environment, Seoul National University, 1 Gwanak-ro, Gwanak-gu, Seoul, South Korea; 4 Youngsan University, College of Nursing, Yangsan Campus, 288 Junam-ro, Yangsan, Gyeongnam 50510 South Korea; 5 Department of Public Health Science at Graduate School of Public Health, Seoul National University, 1 Gwanak-ro, Gwanak-gu, Seoul, South Korea; 6 College of Natural Sciences, Department of Statistics, Seoul National University, 1 Gwanak-ro, Gwanak-gu, Seoul, South Korea; 7 Department of Public Health Science at Graduate School of Public Health, Institute of Health and Environment, Seoul National University, 1 Gwanak-ro, Gwanak-gu, Seoul, South Korea

## Erratum

After the publication of this work [1] it was noticed that the SPIRIT figure template was incorrectly published as Fig. 1. The original version of this article was updated with the correct figure. Please see the correct Fig. 1 below.

**Fig. 1:**
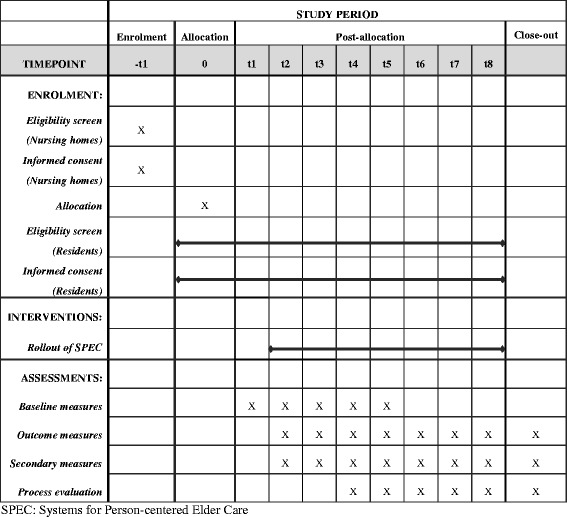
The schedule of enrolment, interventions, and assessments of the SPEC intervention
